# First Pass Effect and Location of Occlusion in Recanalized MCA M1 Occlusions

**DOI:** 10.3389/fneur.2022.884235

**Published:** 2022-05-02

**Authors:** Hisham Salahuddin, Rahul R. Rao, Syed F. Zaidi, Paige Prologo-Richardson, Fatima Khalid, Linda Saju, Muhammad Asif Taqi, Richard R. Burgess, Mouhammad A. Jumaa

**Affiliations:** ^1^Department of Neurology, ProMedica Neurosciences, Toledo, OH, United States; ^2^Department of Neurology, University of Toledo, Toledo, OH, United States; ^3^Department of Neurology, Antelope Valley Hospital, Lancaster, CA, United States; ^4^Department of Neurology, Los Robles Hospital, Thousand Oaks, CA, United States

**Keywords:** large vessel occlusion (LVO), acute ischemic stroke, thrombectomy, first pass effect (FPE), location

## Abstract

**Background:**

The first pass effect has been shown to improve clinical outcomes in patients with middle cerebral artery (MCA) M1 occlusions.

**Objective:**

To determine the rates of first pass effect in MCA M1 occlusions and determine if proximal or distal location of occlusion modified clinical outcomes.

**Methods:**

Patients with recanalized MCA M1 occlusions who underwent endovascular thrombectomy (EVT) were reviewed to determine the effect of first pass effect (FPE) and location of occlusion on clinical outcomes. MCA occlusions were classified as proximal if they included the first two thirds of the MCA and involved the lenticulostriate vessels, or distal if they did not.

**Results:**

We included 261 patients of which 27% achieved FPE. Of the cohort, there were 91 (35%) proximal MCA occlusions and 170 (65%) distal MCA occlusions. Baseline demographics and treatment time metrics were comparable across both groups. There was a trend toward good clinical outcome in patients with or without a FPE (60 vs. 46%; *p* = 0.06), however a higher rate of excellent clinical outcome was noted in patients with FPE (46 vs. 30%; *p* = 0.02). When compared by location, patients with distal MCA occlusions had a higher rate of excellent clinical outcome (40 vs. 25%; *p* = 0.02). Multivariable analysis showed that distal MCA occlusion was the strongest predictor of an excellent clinical outcome and first pass effect.

**Conclusion:**

Patients with MCA M1 occlusions with FPE have a higher rate of excellent clinical outcomes compared to those who did not. Location of MCA occlusion appears to modify rates of first pass effect and excellent clinical outcomes.

## Introduction

Mechanical thrombectomy (MT) is the gold standard treatment for ischemic stroke due to anterior circulation large vessel occlusions (LVO) (1, 2). LVOs comprise up to 20% of all acute ischemic stroke and of these, about 50–65% affect the middle cerebral artery (MCA) (3). While reperfusion is undoubtedly beneficial, studies have shown a significantly beneficial first pass effect when achieving full reperfusion at first attempt compared to subsequent passes (4, 5).

Multiple passes have high risk of endothelial damage, clot fragmentation with distal embolization, and hemorrhagic conversion (1, 6–8). Worse clinical outcomes have been noted in patients with a higher number of passes and lower degree of final reperfusion (9). In contrast, the first pass effect (FPE), defined as Thrombolysis in Cerebral Infarction Scale (TICI) 3, has been shown to have several favorable effects. These outcomes include better functional outcomes at 90 days determined by modified Rankin score from 0 to 2, lower occurrence of embolic events, lower healthcare costs, and lower mortality at 90 days compared to those with multiple passes (1, 6, 7, 10, 11).

Within the MCA territory itself, studies have shown that larger M1 diameter, a higher collateral score, and size of stent-retriever predict a higher rate of first-pass effect (12, 13). However, the FPE is likely modified by other factors such as clot composition and location of occlusion (14). Underlying pathophysiological mechanisms and thrombus composition likely differ in patients with proximal and distal MCA occlusions (15). A comparison of FPE between occlusions of the proximal and distal (sparing lenticulostriate branches) M1 segments of the MCA has yet to be performed.

In this study, we aim to determine the first pass effect in patients with MCA M1 occlusions and compare them to recanalized MCA M1 occlusions without a first pass effect. We also aim to determine if there is a difference in rates of FPE and clinical outcomes in patients with a proximal or distal MCA occlusion.

## Methods

### Study Population

With institutional review board approval, we performed an investigator-initiated retrospective study of patients who underwent successful reperfusion (TICI 2b or greater) for a MCA M1 occlusion at one comprehensive and one thrombectomy capable hospital in Ohio between 2016 and 2020. Patients were identified from a prospectively collected database at both centers. The data that support the findings of this study are available from the corresponding author upon reasonable request. Mechanical thrombectomy was performed by the same operators (SZ, MJ, RB) at both facilities. Patients were excluded from the study if they were younger than 18 years, did not achieve successful reperfusion, or had multifocal intracranial occlusions.

Patients were eligible for mechanical thrombectomy if they had a CT brain with an Alberta Stroke Program Early CT Score (ASPECTS) of 6 or greater, presented within 6 h of last known normal or within 24 h of last known normal with a favorable CT perfusion imaging profile as assessed by the performing neurointerventionalists. Patients who presented within 4.5 h from last seen normal received IV tPA if there were no contraindications.

### Radiological Variables

Baseline demographics (age, sex, vascular risk factors), treatment times, procedural details, admission NIHSS, and modified Rankin Scale (mRS) at baseline were collected in a prospectively maintained database. Two neurointerventionalists (MJ, SZ) independently assessed the location of MCA occlusions on angiographic images as proximal or distal; in case of discrepancy, consensus with a third neurointerventionalist (RB) was obtained. The length of the MCA was defined as the distance from the carotid terminus to the bifurcation point of the middle cerebral artery. Proximal MCA M1 occlusions were defined as those occlusions whose face involved the proximal lenticulostriate vessels and involved the first two thirds of the MCA. Distal occlusions were occlusions of the MCA which involved the distal one third of the MCA and preserved lenticulostriate branches. If concurrent proximal and distal MCA occlusion were identified in a patient, these would be categorized as proximal as the lenticulostriate arteries are not spared. Reperfusion of the MCA territory was assessed using the revised TICI scale (16). A first pass effect was achieved when a single MT attempt resulted in TICI 2c or 3 reperfusion. A modified First Pass Effect (mFPE) was defined as a single MT attempt resulting in TICI 2b reperfusion or greater.

CT brain images were reviewed for hemorrhage and confirmation of ASPECTS by an author (RR) and compared with reports from the neuro-radiologist and neuro-interventionalist, respectively. Intracranial hemorrhage was defined according to ECASS III criteria, and symptomatic intracranial hemorrhage (sICH) was any intracranial hemorrhage resulting in an increase in 4 or more points on the NIHSS (17).

### Outcomes

Ninety day mRS scores were assessed by vascular neurologists during follow up clinic visits who were blinded to the exact location of the MCA occlusion. A good clinical outcome was defined as a mRS score of 0–2, whereas excellent clinical outcome was defined as a mRS score of 0–1.

Mechanical thrombectomy was performed by one of three neurointerventionalists (SZ, MJ, RB) using direct aspiration, use of a stentriever, or a combination of both. Technique of MT was left to the discretion of the treating neurointerventionalists. Procedures were performed under conscious sedation or under general anesthesia in cases of respiratory distress or significant agitation intra-procedurally.

The primary outcome of interest was first pass effect in the overall cohort and secondary outcomes of interest were excellent and good clinical outcomes, proportion of proximal vs. distal MCA occlusions, and hemorrhagic complications.

### Statistical Analysis

Data from the prospectively collected MT database in Microsoft Excel was exported to a statistical analysis software, “R: A language and environment for statistical computing; EZR version 1.32.” Continuous variables were analyzed using the student's *T*-test or Mann Whitney test and categorical data was analyzed using the Fisher Exact test. Multivariate logistic regression analysis was performed to assess for predictors of first pass effect as well as predictors of an excellent clinical outcome. A *p*-value of <0.05 was considered statistically significant. Patients who were lost to follow up were excluded from the final analysis.

## Results

**Figure 1:**
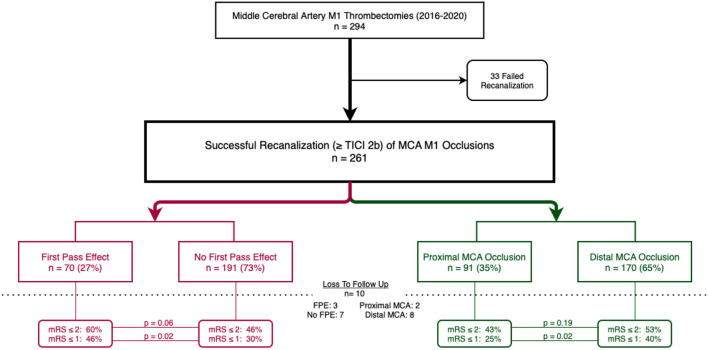
Flow chart of patients included in the study; cohort by first pass effect in red (left) and cohort by proximal or distal MCA occlusions in green (right). Ten patients were loss to follow up.

During the study period, 294 patients with MCA M1 occlusions were identified, of which 33 patients had failed MCA M1 MT. A total of 261 patients underwent successful mechanical thrombectomy for a MCA M1 occlusion were included in our analysis. The cohort consisted of 147 (56.3%) women and the mean age was 70.1 ± 14.4 years. Seventy patients (26.8%) had a first pass effect. FPE was achieved using aspiration only in 43 (61%) patients and a stentriever in the remaining 27 (39%) of patients. Ninety one patients (34.9%) had proximal MCA occlusion, and 170 patients (65.1%) had distal MCA occlusion (see Figure 1; Supplementary Table 1). One hundred and six (41%) patients had a mFPE (Table 1).

**Table 1:** Baseline demographics, procedural characteristics, and clinical outcomes in patients with or without a first pass effect for MCA M1 occlusions.

	**Total**	**FPE (%)** ***n* = 70**	**No FPE (%) *n* = 191**	***P*-value**
**Baseline demographics**
Age	70.1 ± 14.4	71.8 ± 12.2	69.5 ± 15.1	0.51
Female	147 (56.3)	38 (54.3)	109 (57.1)	0.78
Hypertension	208 (79.7)	58 (82.9)	150 (78.5)	0.49
Hyperlipidemia	166 (63.6%)	48 (68.6)	118 (61.8)	0.38
Diabetes mellitus	70 (26.8)	23 (32.9)	47 (24.6)	0.21
Coronary artery disease	73 (28)	22 (31.4)	51 (26.7)	0.44
Atrial fibrillation	124 (47.5)	33 (47.1)	91 (47.6)	1
Smoking	64 (24.5)	17 (24.3)	47 (24.9)	1
ASPECT score^*^	9 (8–9)	9 (8–9)	9 (8–9)	0.06
NIHSS^*^	17 (12–21)	16 (10–21)	17 (13–21)	0.48
IV tPA^*^	82 (31.4)	27 (38.6)	55 (28.8)	0.14
**Procedural characteristics**
Proximal MCA occlusion	91 (34.9)	18 (19.8)	73 (80.2)	0.08
Distal MCA occlusion	170 (65.1)	52 (30.6)	118 (69.4)	
Proximal ICA^*^	20 (7.7)	8 (11.4)	12 (6.3)	0.19
mFPE^*^	106 (40.6)	70 (100)	36 (18.8)	2.2 × 10^−16^
No of passes		1	2.6 ± 1.4	1.3 × 10^−18^
IA tPA	11 (4.2)	1 (1.4)	10 (5.2)	0.30
**Clinical outcome and procedural complications**
Dissection	5 (1.9)	1 (1.4)	4 (2.1)	1
Hemorrhagic transformation	72 (27.6)	15 (21.4)	57 (29.8)	0.21
Parenchymal hematoma	20 (7.7)	4 (5.7)	16 (8.4)	0.60
Excellent clinical outcome	86 (33)	31 (46.3); *n* = 67	55 (29.9); *n* = 184	0.02
Good clinical outcome	124 (47.5)	40 (59.7); *n* = 67	84 (45.7); *n* = 184	0.06
Mortality at 90 days	56 (21.5)	15 (22.4); *n* = 67	41 (22.3); *n* = 184	1
**Time metrics**
Door to groin puncture; median (IQR)	57 (34–87)	56 (33–79)	58 (36–90)	0.65
Door to reperfusion; median (IQR)	79 (43–118)	54 (28–104)	80 (50–124)	0.08
Onset to arrival; median (IQR)	209 (82–487)	247 (114–681)	197 (8–479)	0.24
Procedure time; median (IQR)	40 (22–75)	28 (19–75)	44 (25–72)	0.23

* *ASPECT Score, Alberta Stroke Program Early CT Score; NIHSS, National Institute of Health Stroke Scale; IV tPA, Intravenous tissue plasminogen activator; Proximal ICA, Proximal Internal Carotid Artery (tandem) occlusion; mFPE, Modified First Pass Effect*.

### Baseline Characteristics

There was no significant difference in baseline demographics including age and vascular risk factors in patients who achieved FPE and those who did not. Median ASPECTS [9, (8–9)] and treatment time metrics were comparable across both groups. Rate of intravenous tPA administration (38.6 vs. 28.8%; *p* = 0.14) and median NIHSS [16 (12–21) vs. 17 (10–21); *p* = 0.48] in patients with and without FPE were similar, respectively. Of the cohort, twenty patients (7.7%) had a proximal extracranial internal carotid artery (tandem) occlusion with comparable rates in both proximal and distal groups.

### Outcomes Related to FPE

**Figure 2:**
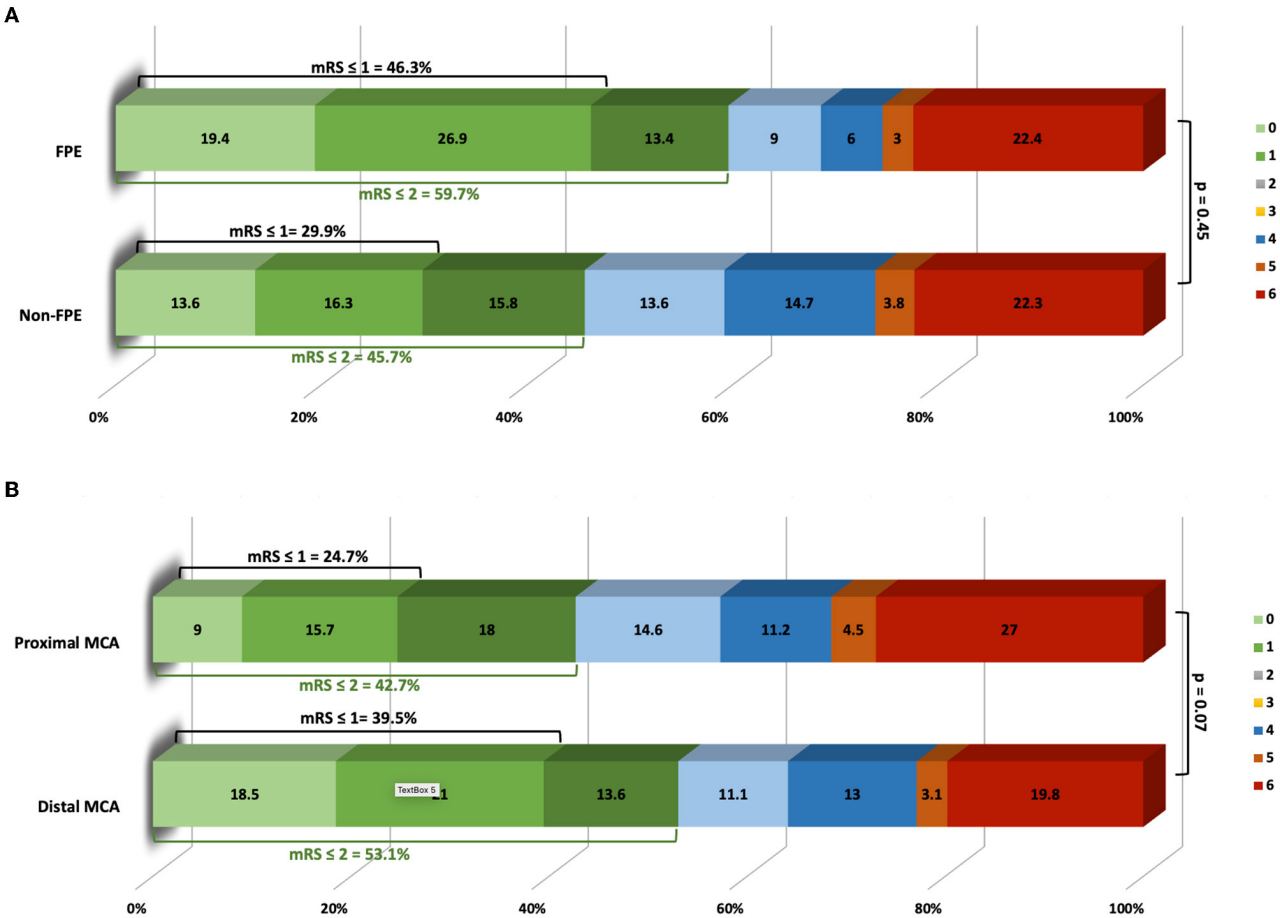
**(A)** Modified Rankin Scale of patients with first pass effect and no first pass effect at 3 months. **(B)** Modified Rankin Scale of patients with proximal or distal middle cerebral artery occlusions at 3 months.

Ten (3.8%) patients were lost to follow up; three patients in the FPE group and 7 patients in the non-FPE group. A good clinical outcome was achieved in forty patients (59.7%) with a first pass effect and eighty-four patients (45.7%) without a first pass effect (*p* = 0.06). Thirty one patients of the sixty-seven with a first pass effect compared to fifty-five of the one hundred and eighty-four patients without FPE achieved excellent clinical outcome at 3 months (46.3 vs. 29.9%; *p* = 0.02). There was no significant different in rates of dissection, hemorrhagic transformation, or parenchymal hematoma between the FPE and non-FPE group. Multivariable logistic analysis after adjusting for age, ASPECTS, and NIHSS showed that distal MCA occlusion (OR 0.48; CI 0.23–1; *p* = 0.049) was the strongest independent predictor of a first pass effect (see Figure 2; Supplementary Table 2).

### Outcomes Related to Occlusion Location

When patients were classified as proximal or distal MCA occlusions, we found that patients with a proximal MCA occlusion had a lower rate of excellent clinical outcome [twenty-two patients (24.7%) vs. sixty-four (39.5%); *p* = 0.02], had a higher number of passes (2.4 ± 1.4 vs. 2.1 ± 1.4; *p* = 0.02), higher NIHSS [18 (14–22) vs. 16 (12–20); *p* = 0.003], and higher rate of hemorrhagic transformation [thirty-four patients (37.4%) vs. forty (23.5%); *p* = 0.02] (see Supplementary Figure 1).

### Multivariate Analysis

In multivariable logistic regression analysis, distal MCA occlusion (OR 0.49; CI 0.25–0.97; *p* = 0.04) was the strongest predictor of an excellent outcome and reached significance while FPE did not (OR 1.9, CI 0.97–3.9; *p* = 0.06) (see Supplementary Table 3).

## Discussion

This retrospective analysis of patients achieving successful reperfusion of middle cerebral artery M1 occlusions revealed a first pass effect of 26.8%. Distal MCA occlusion location was the strongest predictor of a FPE. Furthermore, distal MCA occlusion was also the strongest predictor of an excellent clinical outcome. While previous studies have shown that a first pass effect results in improved clinical outcomes and increases with more distal occlusions (M2 > M1 > TICA), this is the first study to evaluate the first pass effect in patients with proximal or distal M1 MCA occlusions.

Etiological risk factors associated with location of middle cerebral artery occlusions (MCAO) have been shown to differ, indicating that proximal and distal MCAO should be evaluated separately. Chang et al. found that proximal MCAO is more frequently associated with hyperlipidemia and large artery atherosclerosis whereas distal MCAO is more frequently associated with hypertension, atrial fibrillation, and cardioembolic infarcts (15). Additionally, Moyamoya disease has a predisposition for distal internal carotid artery and proximal MCA involvement, indicating that pathophysiology of proximal MCA vessels maybe closer to that of distal ICA than distal MCAs. Underlying intracranial atherosclerotic disease (ICAD) may be a reason why it may be difficult to achieve FPE in these patients. Interestingly, atrial fibrillation was not significantly different between the proximal vs. distal M1 groups (*p* = 0.26). Hypertension, hyperlipidemia, and diabetes mellitus were not significantly different.

Distal MCAOs have been shown to present with lower NIHSS scores, similar to our study, and have improved clinical outcomes and mortality compared with proximal MCAO (18, 19). Location of thrombus in the MCA exhibits a dose-response relationship with clinical outcome at 3 months with both intravenous thrombolysis as well as mechanical thrombectomy (18–20).

Differences in clinical outcomes from proximal or distal MCAO may be attributable to multiple anatomic and physiological factors. Pre-lenticulostriate involvement of clot originating at the proximal end of the M1 segment which supplies the internal capsule and basal ganglia may result in more severe clinical syndromes and a higher NIHSS at presentation. The lack of a developed collateral circulation to the basal ganglia results in shorter time to infarction and lower ASPECTS on presentation. Wider diameter proximal MCAOs suggest larger clot burden in proximal MCAOs which decreases effectiveness of intravenous thrombolysis. Furthermore, occlusion location has also been shown as the strongest predictor of clot length, with proximal MCAOs having clots > 8 mm more frequently (21). Thrombus length has not been shown to impact successful reperfusion rates (22, 23); however a shorter thrombus has been associated with higher rates of FPE in one study (24). Lastly, hemodynamic differences with proximal and distal MCAOs may impact flow dynamics. The impact of occlusion location on FPE has not been well studied, although the TRACK registry did not show an impact of occlusion location on FPE (25). Our data shows that distal MCAOs have numerically higher rate of FPE (30.6 vs. 19.8%; *p* = 0.08) and a statistically significant higher rate of excellent clinical outcomes (39.5 vs. 24.7%; *p* = 0.02).

The first pass effect has been shown to impact clinical outcomes and mortality in patients undergoing mechanical thrombectomy (4). The FPE rate in our study was 26.8%, which is comparable to rates seen in the ASTER trial (28.9%) and TRACK registry (23%) (25, 26). While FPE is an important predictor of clinical outcome, it is also likely a surrogate for a multitude of factors influencing final clinical outcome including disease severity, collateral status, clot integrity and risk of distal embolization, efficiency of MT devices, and technique of MT (12, 27). Rates of excellent clinical outcomes were significantly higher in the FPE group compared to the non-FPE group. Furthermore, our study shows that distal MCAO is the strongest predictor of FPE in patients with MCAOs and a strong predictor of achieving an excellent clinical outcome.

In this study, we show that FPE is significantly impacted by proximal or distal location of middle cerebral artery occlusions. While FPE of MCAO results in improved clinical outcomes, the true impact of FPE for MCAO may be exaggerated by combining proximal and distal MCAOs. While our study could not demonstrate a statistically significant difference for distal vs. proximal MCAO rates of FPE, further studies are needed to evaluate if a difference between FPE for proximal or distal occlusions are present and if the rate of FPE for distal occlusions are inherently higher.

Strengths of this study include assessment of FPE in only recanalized patients and independent assessment of thrombus location by experienced neurointerventionalists. However, our study suffers from several limitations including the lack of core lab assessment of MCAOs, lack of collateral assessment, lack of standardized mechanical thrombectomy technique (aspiration, stentriever, or combined), and long duration of study. This may result in over estimation of first pass effect seen in distal MCA occlusions. This is also a single center study. We did not record baseline mRS as a demographic variable as forty-five patients were missing this data point. Further limitations include lack of differentiation between embolic and atherosclerotic occlusions. Thrombus type is known to play a role in disease severity and reperfusion rates. We also did not identify patients whose lenticulostriate supply may be different than the proximal M1 segment which may also lead to differences in radiographic and clinical outcomes (19). We did use a similar classification of proximal vs. distal M1 occlusion as seen in a recent study (28). Our study did not compare thrombus location on non-invasive imaging to angiographic images and did not account for possible thrombus migration prior to onset to mechanical thrombectomy. Lastly, our study suffers from inherent bias associated with retrospective studies.

In conclusion, patients with MCAOs had a first pass effect of 26.8% which resulted in an increase in excellent clinical outcome at 3 months and a numerically higher rate of good clinical outcome compared to patients without FPE. Location of thrombus in the middle cerebral artery was a strong predictor of first pass effect and an excellent clinical outcome. Larger, prospective studies are needed to evaluate the impact of MCAO location and first pass effect.

## Data Availability Statement

The raw data supporting the conclusions of this article will be made available by the authors, without undue reservation.

## Author Contributions

HS: contributed toward data gathering, statistical analysis, and writing of article. RR: contributed toward data gathering and writing of article. SZ and RB: contributed toward data gathering and conceptualization. PP-R, FK, and LS: contributed toward data gathering. MT: contributed toward statistical analysis. MJ: contributed toward conceptualization, data gathering, and writing of article. All authors contributed to the article and approved the submitted version.

## Conflict of Interest

The authors declare that the research was conducted in the absence of any commercial or financial relationships that could be construed as a potential conflict of interest.

## Publisher's Note

All claims expressed in this article are solely those of the authors and do not necessarily represent those of their affiliated organizations, or those of the publisher, the editors and the reviewers. Any product that may be evaluated in this article, or claim that may be made by its manufacturer, is not guaranteed or endorsed by the publisher.

## Abbreviations

FPE: First-pass effect
MCAO: middle cerebral artery occlusion.
