# Supplementary material for: First Pass Effect and Location of Occlusion in Recanalized MCA M1 Occlusions
Source: Front Neurol. 2022 May 2;13:884235. doi: 10.3389/fneur.2022.884235 (PMC9108282; doi:10.3389/fneur.2022.884235)

Supplemental Table 1: Baseline demographics, procedural characteristics, and clinical outcomes in patients with proximal or distal MCA M1 occlusions

|  | Proximal  (n=91) | Distal  (n=170) | P-Value |
| --- | --- | --- | --- |
| **Baseline Demographics** |  |  |  |
| Age | 68.8 ± 14.8 | 71.0 ± 14.2 | 0.20 |
| Female | 41 (45.1) | 106 (62.4) | 0.009 |
| Hypertension | 71 (78) | 137 (80.6) | 0.07 |
| Hyperlipidemia | 56 (61.5) | 110 (64.7) | 0.69 |
| Diabetes Mellitus | 22 (24.2) | 48 (28.2) | 0.56 |
| Coronary Artery Disease | 27 (29.7) | 46 (27.1) | 0.67 |
| Atrial Fibrillation | 48 (52.7) | 76 (44.7) | 0.24 |
| Smoking | 24 (26.4) | 42 (24.7) | 0.77 |
| ASPECT Score* | 8.5 (8-9) | 9 (8-9) | 0.02 |
| NIHSS* | 18 (14-22) | 16 (12-20) | 0.003 |
| IV tPA* | 24 (26.4) | 58 (34.1) | 0.21 |
| **Procedural Characteristics** |  |  |  |
| mFPE* | 29 (31.9) | 77 (45.3) | 0.047 |
| FPE* | 18 (19.8) | 52 (30.6) | 0.08 |
| No of Passes | 2.4 ± 1.4 | 2.1 ± 1.4 | 0.02 |
| **Clinical Outcome & Procedural Complications** |  |  |  |
| Dissection | 3 (3.3) | 2 (1.2) | 0.34 |
| Hemorrhagic Transformation | 34 (37.4) | 40 (23.5) | 0.02 |
| Parenchymal Hematoma | 11 (12.1) | 9 (5.3) | 0.08 |
| Excellent Clinical Outcome | 22 (24.7)  n=89 | 64 (39.5)  n=162 | 0.02 |
| Good Clinical Outcome | 38 (42.7)  n=89 | 86 (53.1)  n=162 | 0.19 |
| Mortality at 90 days | 24 (27)  n=89 | 32 (19.8)  n=162 | 0.16 |
| **Time Metrics** |  |  |  |
| Door to Groin Puncture; median (IQR) | 55 (34-80) | 58 (35-94) | 0.40 |
| Door to Reperfusion; median (IQR) | 79 (57-132) | 76 (37-110) | 0.25 |
| Onset to Arrival; median (IQR) | 213 (81-368) | 192 (92-611) | 0.71 |
| Procedure Time; median (IQR) | 40 (24-67) | 41 (22-78) | 0.93 |
| *ASPECT Score: Alberta Stroke Program Early CT Score, NIHSS: National Institute of Health Stroke Scale, IV tPA: Intravenous tissue plasminogen activator, mFPE: Modified First Pass Effect, FPE: First Pass Effect | | | |

Supplemental Table 2: Multivariable analysis for predictors of a first pass effect

|  | Odds Ratio | Lower 95% CI | Upper 95% CI | P-Value |
| --- | --- | --- | --- | --- |
| **Distal MCA** | **0.48** | **0.226** | **0.99** | **0.049** |
| ASPECT Score | 1.19 | 0.879 | 1.62 | 0.259 |
| Age | 1.01 | 0.98 | 1.03 | 0.490 |
| NIHSS | 0.99 | 0.946 | 1.05 | 0.933 |

Supplemental Table 3: Multivariable analysis for predictors of an excellent clinical outcome

|  | Odds Ratio | Lower 95% CI | Upper 95% CI | P-Value |
| --- | --- | --- | --- | --- |
| **ASPECT Score** | **1.4** | **1.05** | **1.88** | **0.022** |
| **Distal MCA** | **0.492** | **0.249** | **0.972** | **0.041** |
| FPE | 1.94 | 0.97 | 3.88 | 0.061 |
| Age | 0.983 | 0.961 | 1.0 | 0.121 |

Supplemental Figure 1: Bar chart showing higher rates of excellent clinical outcome in the distal MCA group with FPE compared to proximal MCA group with FPE.


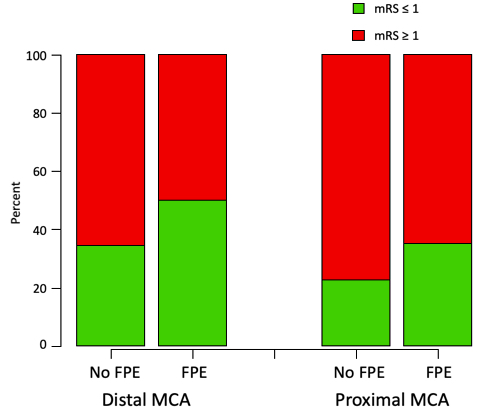

Supplement: Supplementary file 1 [file Data_Sheet_1.docx]
